# Supplementary material for: Preventing the Collapse Behavior of Polyurethane Foams with the Addition of Cellulose Nanofiber
Source: Polymers (Basel). 2023 Mar 17;15(6):1499. doi: 10.3390/polym15061499 (PMC10058122; doi:10.3390/polym15061499)
Supplement: Supplementary file 1 [file polymers-15-01499-s001.zip › polymers-2273456-supplementary.pdf]

polymers

MDPI

Article

Preventing the Collapse Behavior of Polyurethane Foams with the Addition of Cellulose Nanofiber

Sanghyeon Ju [1,2], Ajeong Lee [1], Youngeun Shin [1], Hyekyeong Jang [1], Jin-Woo Yi [1], Youngseok Oh [1], Nam-Ju Jo [2,*] and Teahoon Park [1,*]

1 Composites Research Division, Korea Institute of Materials Science (KIMS), 797, Changwon-Daero, Seongsan-Gu, Changwon-si 51508, Republic of Korea
2 School of Chemical Engineering, Pusan National University, Busan 46241, Republic of Korea
* Correspondence: namjujo@pusan.ac.kr (N.-J.J.); thpark@kims.re.kr (T.P.); Tel.: +82-055-280-3158 (T.P.)

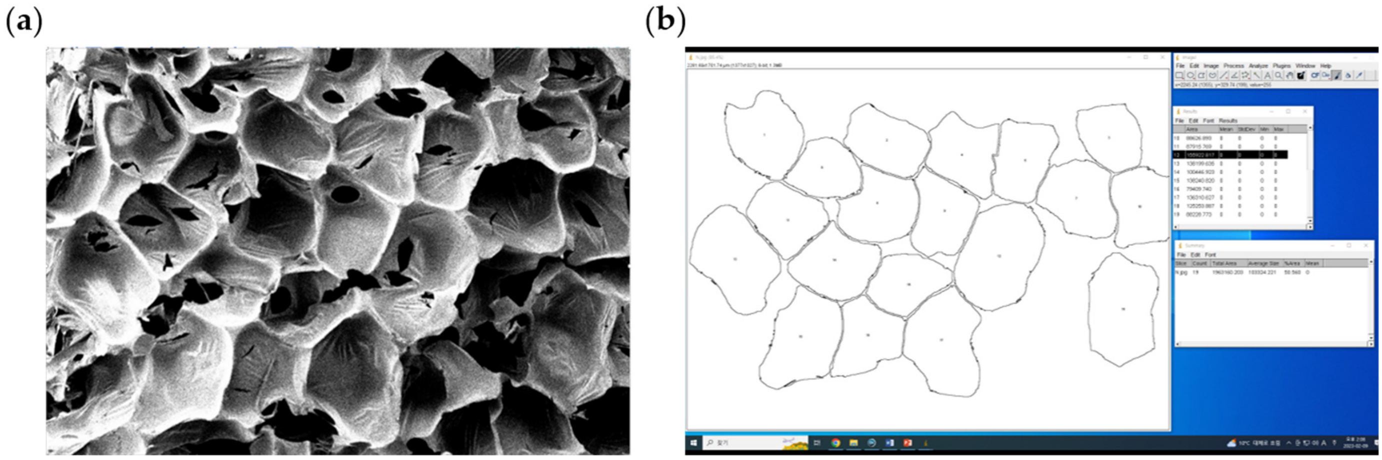

Figure S1. (**a**) The SEM of N, (**b**) Capture of measuring average cell area of N using Image J.

Figure S1 is an example of measuring the average cell area of samples using Image J freeware. Measurements were conducted without counting cells that could not be considered cells.

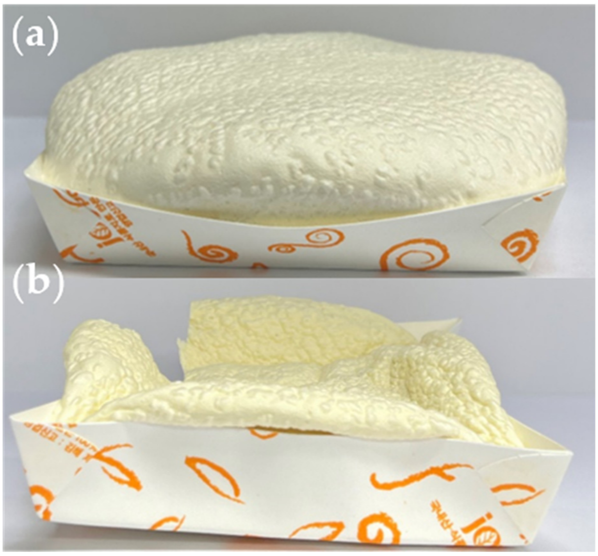

Figure S2. The picture of C3 at (**a**) day 1 and (**b**) day 14.

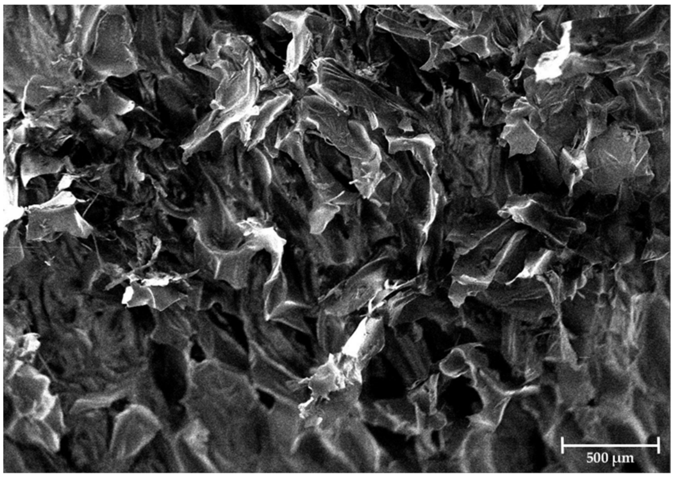

Figure S3. The SEM image of collapsed sample N.
